# A Facile, One-Step Synthesis of Silicon/Silicon Carbide/Carbon Nanotube Nanocomposite as a Cycling-Stable Anode for Lithium Ion Batteries

**DOI:** 10.3390/nano9111624

**Published:** 2019-11-15

**Authors:** Yi Zhang, Kai Hu, Yunlei Zhou, Yingbin Xia, Nengfei Yu, Guanglei Wu, Yusong Zhu, Yuping Wu, Haibo Huang

**Affiliations:** 1 School of Energy Sciences and Engineering, Nanjing Tech University, Nanjing 211816, China; zhangy@njtech.edu.cn (Y.Z.); 201861108005@njtech.edu.cn (K.H.); leslie@njtech.edu.cn (Y.X.); yunf@njtech.edu.cn (N.Y.); wuyp@njtech.edu.cn (Y.W.); 2 College of Engineering and Applied Sciences, Nanjing University, Nanjing 210093, China; zylnju@smail.nju.edu.cn; 3 Institute of Materials for Energy and Environment, State Key Laboratory of Bio-Fibers and Eco-Textiles, College of Materials Science and Engineering, Qingdao University, Qingdao 266071, China; wuguanglei@mail.xjtu.edu.cn; 4 School of Mechanical and Electric Engineering, Jiangsu Provincial Key Laboratory of Advanced Robotics, & Robotics and Microsystems Center, Soochow University, Suzhou 215123, China

**Keywords:** silicon, carbon nanotube, magnesium thermal reduction, anode

## Abstract

Silicon/carbon nanotube (Si/CNTs) nanocomposite is a promising anode material for lithium ion batteries (LIBs). Challenges related to the tricky synthesis process, as well as the weak interaction between Si and CNTs, hinder practical applications. To address these issues, a facile, one-step method to synthesize Si/CNTs nanocomposite by using silica (SiO_2_) as a reactant via a magnesium reduction process was developed. In this synthesis, the heat released enables the as-obtained Si to react with CNTs in the interfacial region to form silicon carbide (SiC). By virtue of the unique structure composed of Si nanoparticles strongly anchored to conductive CNTs network with stable Si–C chemical bonding, the Si/SiC/CNT nanocomposite delivers a stable capacity of ~1100 mAh g^−1^ and a capacity retention of about 83.8% after 200 cycles at a current density of 100 mA g^−1^. Our studies may provide a convenient strategy for the preparation of the Si/C anode of LIBs.

## 1. Introduction

Lithium-ion batteries have the advantages of high energy density, relatively low weight, and retaining power after being recharged hundreds of times. Therefore, they are well established in cameras, watches, mobile devices, and especially electric vehicles (EVs) [1,2,3,4]. However, energy density remains a big obstacle to speeding up the global adoption of lithium ion batteries (LIBs) in EVs. LIBs consist of an arrangement of different layers. The cathode and anode are the two electrodes of the battery, which are essential parts of LIBs. In this reason, there are many research groups worldwide developing new materials replacing the commercial graphite anode in order to improve the LIBs’ energy density [5,6,7,8,9]. Silicon—the second most abundant element in the earth’s crust—shows great promise for Li-ion batteries because it is abundant, and it shows much higher theoretical capacity (4200 mAh g^−1^) than the graphite (372 mAh g^−1^) in current LIBs. However, the charging and discharging process leads to an enormous change in volume of the silicon, and therefore, to a rapid mechanical and electrochemical destruction of the material, and thus, to cell failure [10,11,12]. 

To address the issues, researchers have formed silicon into nano-scale particles [13,14,15], tubes [16,17], wires [18], and films [19] to help accommodate its volume expansion and contraction. For further improvement, the nano-scale Si is often embedded in a matrix, such as amorphous carbon [20,21,22], carbon nanotubes (CNTs) [23,24], or graphene [25], which not only accommodates the severe volume change, but also facilitates transporting electrons. CNTs hold great potential for constructing advanced Si-based anodes because of their high electrical conductivity, high aspect ratio, and high mechanical strength. However, the nature of adhesion between Si and sp^2^ carbons of CNTs is inherently weak. The Si nanoparticles in Si/CNTs nanocomposite are easy to detach from CNTs’ surfaces, resulting in capacity loss.

In order to stabilize Si nanoparticles on CNTs’ surface, silicon carbide (SiC) has been introduced between Si nanoparticles and CNTs. Niu et al. [26] prepared a Si/SiC/CNT nanocomposite which showed improved electrochemical performance. However, the synthesis of SiC needs to be conducted at a high temperature (above 1100 °C), limiting its application and commercialization. The magnesium reduction of silica is a promising method to prepare Si, as it is done at a low temperature [27]. Magnesium’s thermal reduction can enable the as-prepared Si react with carbon to form SiC because magnesium’s thermal reduction is a high exothermic reaction. In this work, we synthesized Si/SiC/CNTs nanocomposite using a thermal reduction of magnesium. A partial reaction between as-prepared Si nanoparticles and CNTs occurred during the magnesium reduction process, forming an interfacial SiC layer which could fix Si onto CNTs. Thus, the Si/SiC/CNT nanocomposite shows excellent cycling stability.

## 2. Experimental

### 2.1. Preparation of Si/SiC/CNT Nanocomposite

**Figure 1:**
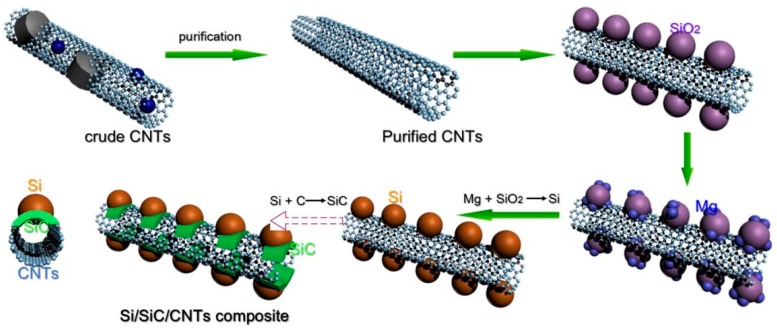
The schematic illustration of Si/SiC/carbon nanotubes’ (CNTs) nanocomposite preparation.

CNTs (Chengdu Alpha Nano Technology Co., Ltd, Chengdu, Sichuan, China) were stirred in nitric acid (5.0 mol L^−1^) for 0.5 h and refluxed at 110 °C for 8 h. After purification treatment, the samples were rinsed and dried under vacuum, and the purified CNTs were obtained. Purified CNTs and SiO_2_ (20 nm, Sigma-Aldrich, St. Louis, MO, USA) at molar ratio of 5:2 were added to ethanol (Sigma-Aldrich, St. Louis, MO, USA) solution, and were sonicated for 12.0 h. Then, the mixture was filtered, washed with ethanol, and freeze-dried overnight. Finally, SiO_2_/CNTs composite was obtained. SiO_2_/CNTs composite and Mg powder (3 μm, Sigma-Aldrich, St. Louis, MO, USA) at a molar ratio of 2:5 (SiO_2_:Mg) were grinded evenly under an argon atmosphere and placed in a corundum boat. The corundum boat was placed into a tube furnace, followed by heating to 650 °C at a ramp rate of 5 °C min^−1^ under an argon gas flow. After reacting at 650 °C for 5.0 h, the sample was purified in HCl solution (1.0 mol L^−1^) for 24.0 h, and then in 1.0 mol L^−1^. HF solution for 0.5 h to dissolve MgO, Mg_2_Si, and SiO_2_. The resultant product was filtered, rinsed, and dried at 100 °C to obtain pure Si/SiC/CNT nanocomposite. The schematic illustration of preparation of Si/SiC/CNT nanocomposite is shown in Figure 1.

### 2.2. Characterization

The structures of crude CNTs, purified CNTs, and Si/SiC/CNT were analyzed by X-ray diffractometry (XRD, D-3, Rigaku Inc., Tokyo, Japan) with Cu Kα radiation and a Raman spectroscope (RE01, Renishaw Inc., London, UK) equipped with a 514 nm diode laser. The morphologies of crude CNTs, purified CNTs, and Si/SiC/CNT were observed by scanning electron microscope (SEM, Quanta 250, FEI Inc., Hillsboro, OR, USA). A Tecnai G2 high-resolution transmission electron microscope (HRTEM, Philips Inc., Amsterdam, Holland), operating at a 100 kV accelerating voltage, and selected area electron diffraction (SAED), were used to analyze the inter-morphologies of crude CNTs, purified CNTs, and Si/SiC/CNT. Thermogravimetric analysis (TGA) of Si/SiC/CNT was obtained by a Thermal Analysis TG/DTA 6300 instrument (NSK Inc., Tokyo, Japan) under O_2_ atmosphere with a heating rate of 5 °C min^−1^.

### 2.3. Electrochemical Measurements

The working electrodes were prepared by dispersing 80 wt% Si/SiC/CNT nanocomposite, 10 wt% carbon black, and 10 wt% polyacrylic acid (PAA, Mw = 150,000) in deionized water to form a uniform slurry. The slurry was coated on a Cu foil and punched into 14 mm diameter disks. Celgard 2400 membrane was used as the separator. The electrolyte was LiPF_6_ (1.0 mol L^−1^) in a mixture of dimethyl carbonate (DMC) and ethylene carbonate (EC) (1:1, *v*/*v*). All cells were tested within a voltage range between 0.01 and 3.0 V. The cycling performances were tested with a battery test system (CT2001A, Wuhan Land Electronic Co. Ltd, Wuhan, Hubei, China). Cyclic voltammetry (CV) curves were recorded on a PGSTAT 302N electrochemical station (Wuhan Land Electronic Co., Ltd., Wuhan, Hubei, China).

## 3. Results and Discussion

The CNTs in this study were produced by chemical vapor deposition (CVD) method. They contained many impurities which significantly affect the physico-chemical properties of CNTs. Therefore, the CNTs needed to be purified. In our study, acid treatment was used to remove the impurities of CNTs. 

**Figure 2:**
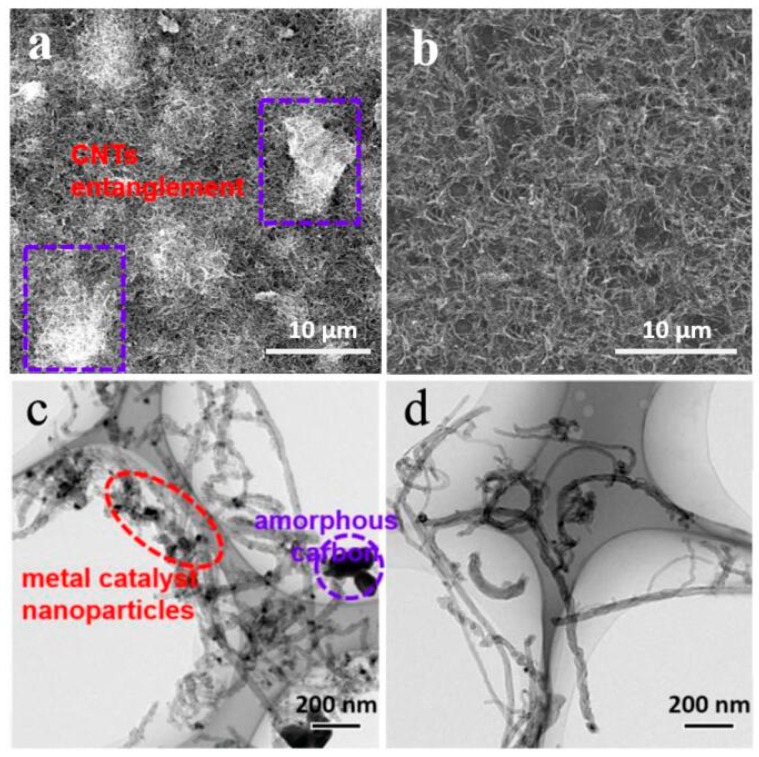
SEM images of crude CNTs (**a**) and purified CNTs (**b**), and TEM images of crude CNTs (**c**) and purified CNTs (**d**).

To test the validity of acid treatment for CNTs, the morphologies and structures of CNTs before and after purification were characterized, as shown in Figure 2. The bright chunks in Figure 2a are agglomerated carbon nanotubes which resulted from existing amorphous carbon impurities on CNTs. After acid treatment, CNTs showed well-separated morphology (Figure 2b). Further evidence of successful removal of impurities comes from the TEM images. Some dark lumps are amorphous carbon and dark spots representing metal catalyst nanoparticles in Figure 2c. No impurities were observable after acid treatment (in Figure 2d).

**Figure 3:**
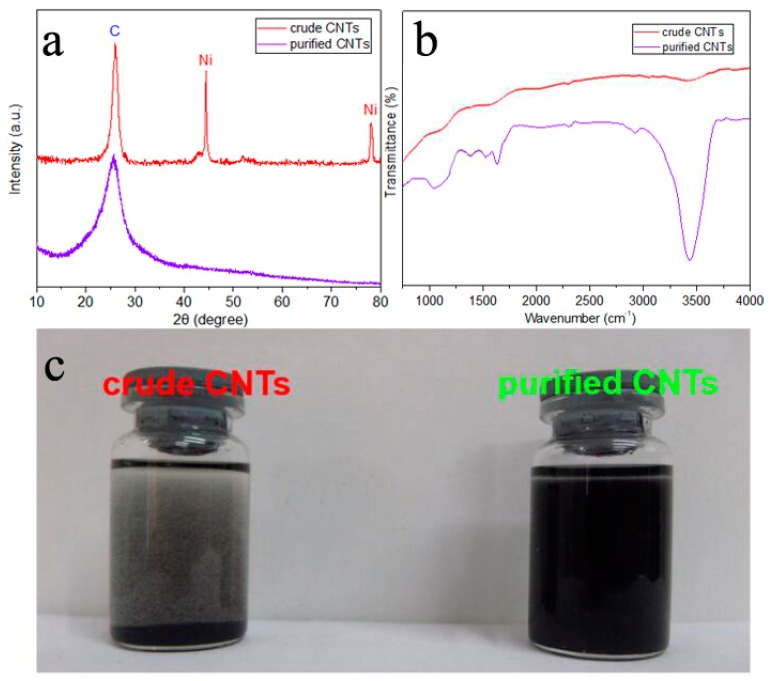
(**a**) XRD patterns of crude CNTs and purified CNTs; (**b**) FTIR spectrum of crude CNTs and purified CNTs; (**c**) the pictures of crude CNTs and purified CNTs dispersed in ethanol.

XRD was used to analyze the structural changes of CNTs. A sharp peak at 2θ = 26.5° can be assigned to (002) plane of CNTs [28]. Two peaks at 44.6° and 76.5° correspond to the (111) plane and (220) plane of Ni (JCPDS, file number 65-2865) [29], which means that the metal catalyst residual in crude CNTs is metal Ni. After acid treatment, the peaks assigned to the metal catalyst disappeared, which indicates that the metal catalysts were eliminated (Figure 3a). The Flourier transformed Infrared (FTIR) results show that a new, strong peak at 3440 cm^−1^ appeared in purified CNTs, which is attributable to the –OH groups on purified CNTs’ surfaces (Figure 3b). Because impurities were removed and the CNTs were simultaneously chemically modified, the purified CNTs can disperse well in ethanol (Figure 3c). These results indicate that the purification treatment is effective.

**Figure 4:**
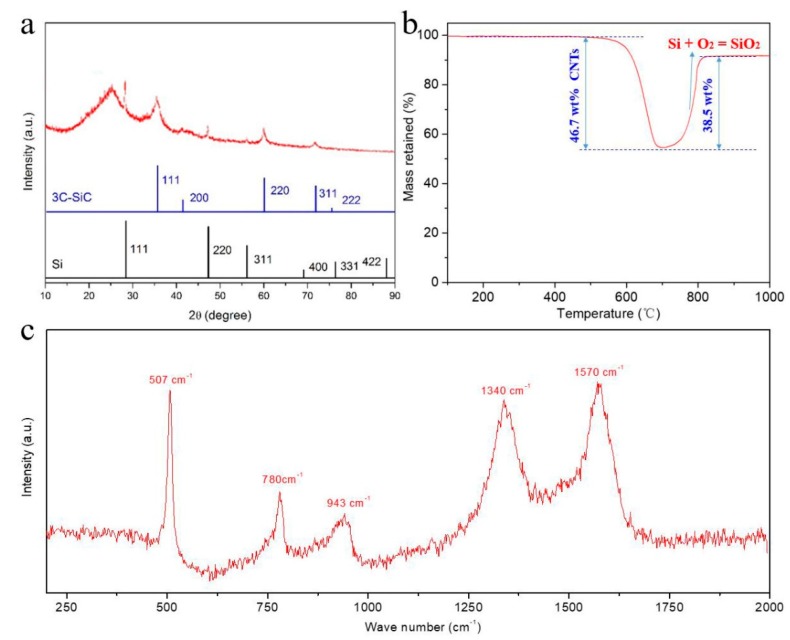
(**a**) XRD patterns of the Si/SiC/CNT nanocomposite; (**b**) TGA of Si/SiC/CNT nanocomposite; (**c**) Raman spectrum of Si/SiC/CNT nanocomposite.

Figure 4a shows XRD pattern of the Si/SiC/CNT nanocomposite. The pronounced peak at 26.5° corresponds to the (002) plane of CNTs. The peaks at about 28.4°, 47.3°, 56.1° and 69.1° can be indexed as the (111), (220), (311), and (400) planes of cubic phase silicon (JCPDS number 27-1402) [30], respectively. The average crystallite size of Si nanoparticles was approximately 20 nm calculated by Debye Scherrer Equation [31] (D = K*λ/β*cosθ, where D is average crystallite size, λ is the X-ray wave length, K is the sharp factor, β is the full-width of half-maximum of the peak, and θ is the diffraction angle). It is worth noticing that group peaks at 35.7°, 41.4°, 60.0°, and 71.8° are in good agreement with the (111), (200), (220), and (311) reflections of 3C-SiC (JCPDS number 65-0360) [32], respectively. In magnesium thermal reduction, Si nanoparticles were obtained by magnesium react with SiO_2_ (2Mg + SiO_2_ = Si + 2MgO). Meanwhile, the as-obtained Si reacted with CNTs in interfacial regions and was partially converted to SiC (Si + C = SiC). The Mg thermal reduction in this research occurred at about 650 °C, which is lower than the temperature (1100 °C) for direct reaction of C with Si. But the magnesium thermal reduction is a highly exothermic reaction. The energy released can heat the surrounding atmosphere to the required temperature of direct reaction between CNTs and Si, resulting in SiC’s generation. To test each component’s content of Si/SiC/CNT nanocomposite, we conducted quantitative phase analysis using Total Pattern Solution (TOPAS software, Bruker Inc., Karlsruhe, Baden-Wurttemberg, Germany). The results showed that Si/SiC/CNT nanocomposite contained 47.1 wt% CNTs, 34.4 wt% Si, and 18.5 wt% SiC. The quantitative compositions of Si/SiC/CNT nanocomposite were confirmed by TGA (Figure 4b). As shown in Figure 4b, the residual percentage weight of Si/SiC/CNT nanocomposite was 53.3 wt% at 700 °C. Thus, the content of CNTs in Si/SiC/CNT nanocomposite was estimated to be 46.7 wt%. The weight of Si/SiC/CNT nanocomposite increases at high temperatures owing to the reaction between Si and O_2_ to form SiO_2_. According to the final remaining weight, the content of Si in Si/SiC/CNT nanocomposite was determined to be 33.7 wt%. TGA analysis showed that the Si/SiC/CNT nanocomposite consisted of 46.7 wt% CNTs, 33.7 wt% Si, and 19.6 wt% SiC, all of which are consistent with the results from TOPAS analysis. The further evidence for the formation of the Si/SiC/CNT nanocomposite was given from the Raman pattern (Figure 4c). The peaks at 1340 cm^−1^ and 1570 cm^−1^ correspond to the D-band (defect) and G-band of CNTs, respectively. The peaks around 507 cm^−1^ and 931 cm^−1^ are typical features of silicon vibration mode, associated with first-order optical phonon (TO) mode and two transverse optical phonons (2TO) mode [33], respectively. The peak at 780 cm^−1^ was from SiC, and related to TO mode of 3C-SiC [34]. 

**Figure 5:**
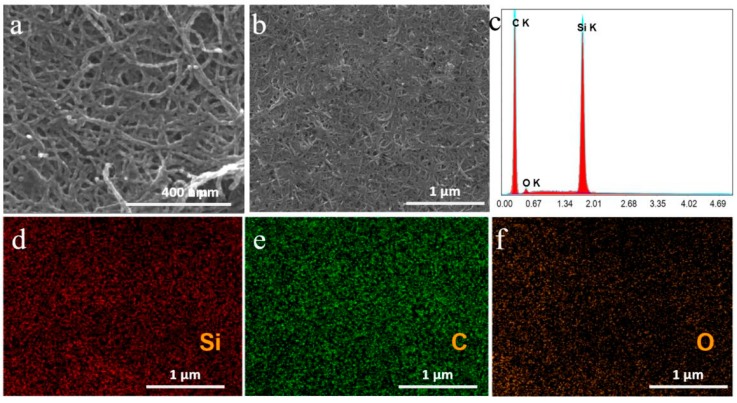
(**a**,**b**) SEM images of Si/SiC/CNT nanocomposite; (**c**) EDX spectrum of Si/SiC/CNT nanocomposite. Element mappings of Si/SiC/CNT nanocomposite: Si (**d**); C (**e**); O (**f**).

SEM images of the Si/SiC/CNT nanocomposite are shown in Figure 5a,b. The Si nanoparticles obtained were uniformly coated on the CNTs’ surface. The diameter of all Si nanoparticles was 20 nm. In our paper, the magnesium thermal reduction contains the following reactions (2Mg + SiO_2_ = Si + 2MgO; 2Mg + Si = Mg_2_Si; C + Si = SiC). There were some impurities (MgO, Mg_2_Si, and SiO_2_) in the Si/SiC/CNT nanocomposite. In order to remove the impurities, the composite was purified in HCl solution (1.0 mol L^−1^ for 24.0 h, and then in 1.0 mol L^−1^ HF solution for 0.5 h. To confirm the purity of the Si/SiC/CNT nanocomposite, an energy dispersive X-ray (EDX) spectrum was obtained and EDX mapping analysis was conducted. Figure 5d–f shows element mappings of Si/SiC/CNT nanocomposite. Uniform C, Si, and O elemental distributions are clearly observable. Figure 5c shows the EDX spectrum of Si/SiC/CNT nanocomposite. Two strong peaks were assigned to C and Si, while a weak peak belongs to O. No Mg peaks appeared which indicates that impurities were removed. The composition of Si/SiC/CNT nanocomposite was calculated from EDX analysis. The results showed that the Si/SiC/CNT nanocomposite contained 52.1 wt% C, 46.7 wt% Si, and 1.2 wt% O.

**Figure 6:**
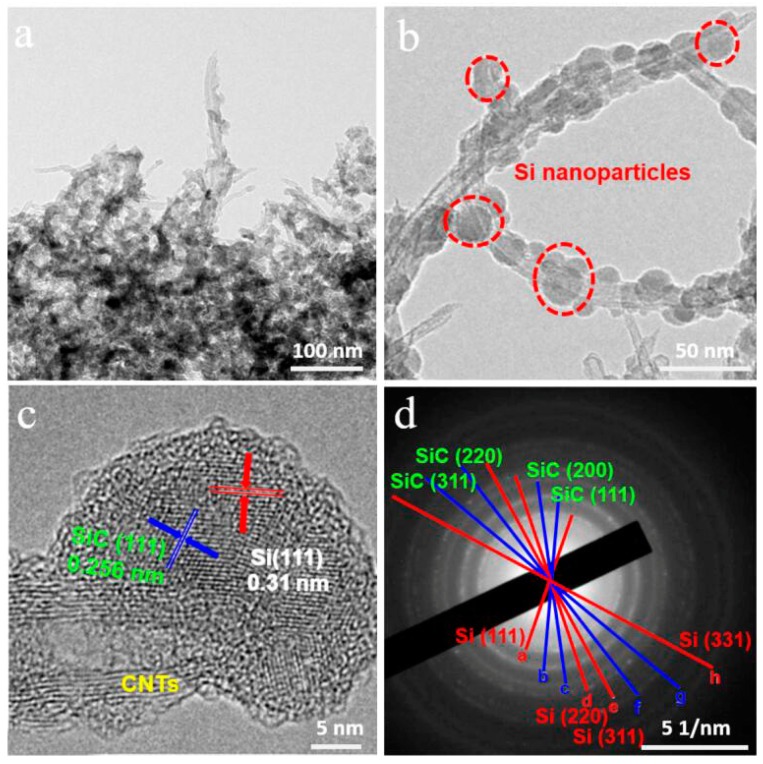
(**a**,**b**) TEM images of Si/SiC/CNT nanocomposite; (**c**) HRTEM image of Si/SiC/CNT nanocomposite; (**d**) SAED pattern of Si/SiC/CNT nanocomposite.

To investigate the inter structure of the Si/SiC/CNT nanocomposite, we conducted a TEM experiment. From Figure 6a, we can observe that many dark spots were distributed on the sidewalls of CNTs. The dark spots represent Si nanoparticles. Figure 6b shows that the Si nanoparticles were half-embedded into CNTs due to consuming CNTs’ carbon atoms to react with Si nanoparticles to generate SiC. To identify each phase, the d-spacing was measured from Figure 6c. Figure 6c demonstrates that the lattice fringe distance in the outer layer was 0.31 nm, which is consistent with the (111) plane of Si. The lattice fringe distance of the middle layer was 0.256 nm, which corresponds to the (110) crystal plane of SiC. The SAED pattern corresponds to (111), (200), (220), and (311) planes of SiC, and (111), (220), (331), and (311) planes of Si (Figure 6d). The results suggest that SiC forms between Si and CNTs.

**Figure 7:**
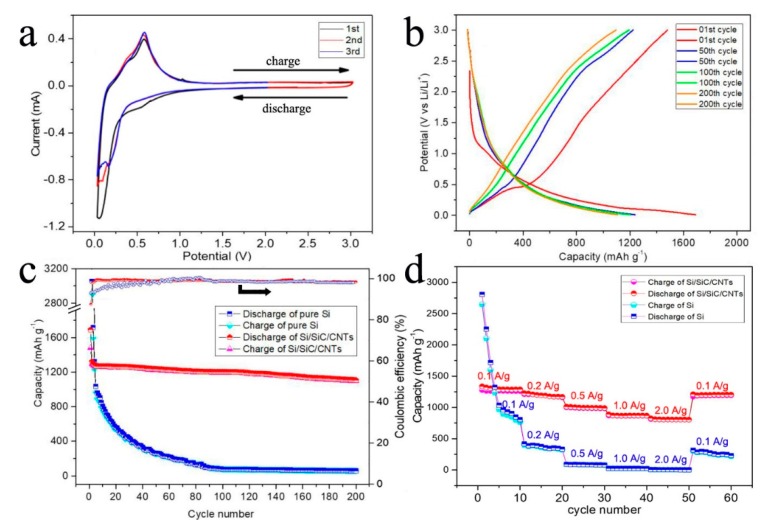
(**a**) Galvanostatic charge/discharge profiles of Si/SiC/CNT nanocomposite; (**b**) CV on Si/SiC/CNT nanocomposite electrode at the scan rate of 0.2 mV s^−1^; (**c**) cycling performance and Coulombic efficiency of pure Si and Si/SiC/CNT nanocomposites; (**d**) rate performances of Si/SiC/CNT nanocomposite and pure Si.

Electrochemical performances of the Si/SiC/CNT nanocomposite are shown in Figure 7a–d. The lithium ion intercalation/deintercalation property of the Si/SiC/CNT anode is depicted by its initial three CV curves with the potentials ranging from 0 to 3.0 V at 0.1 mV s^−1^ (Figure 7a). During the first cycle, four reactions occurred. One peak at 0.14 V is related to formation of Li_x_Si. Two peaks at 0.20 and 0.54 V are attributable to the extraction of Li^+^ from a Li–Si alloy [35]. The small peak located at 0.7 V corresponds to the formation of solid electrolyte interface (SEI). In the next two cycles, the peak at 0.7 V disappeared, which means the SEI was formed. Figure 7b shows the discharge–charge curves of the Si/SiC/CNT nanocomposite electrode after the 1st cycle, 50th cycle, 100th cycle, and 200th cycle at 100 mA g^−1^ in a voltage range of 0.01–3.0 V versus Li/Li^+^. The first discharge curve shows long plateaus at around 0.15 V due to the reaction of Si with Li to form Li_x_Si. After the first cycle, the discharge-charge curves indicate sloping curves, corresponding to Li alloying with Si [36].

Figure 7c shows the cycling stability of the Si/SiC/CNT nanocomposite electrode and pure silicon at a current density of 100 mA g^−1^. The reversible capacity of pure Si electrode was 2252.5 mAh g^−1^ in the initial cycle. After 200 cycles, its reversible capacity decreased to 57.3 mAh g^−1^; only 2.5% of its initial capacity was retained, suggestive of severe volume changes of Si nanoparticles. Si/SiC/CNT nanocomposite showed an initial capacity of 1690.2 mAh g^−1^ with a Coulombic efficiency of 87.6%. The irreversible capacity in the first cycle was due to formation of SEI film. After three cycles, the Coulombic efficiency was higher than 99%, indicating a good reversibility of Si/SiC/CNT nanocomposite. Si/SiC/CNT nanocomposite showed high cyclability with a slight decrease of reversible capacity from 1321.8 to 1107.6 mAh g^−1^ at 100 mA g^−1^ after 200 cycles, and a capacity retention of about 83.4%; those values are higher than those of other similar Si/C anodes [37,38,39]. The rate capability of Si/SiC/CNT nanocomposite is also good (Figure 7d). At current densities of 100, 200, 500, 1000, and 2000 mA g^−1^, the Si/SiC/CNT electrode retained capacities of 1311.1, 1193.5, 996.4, 869.3, and 807.6 mAh g^−1^. After the current density returned to 100 mA g^−1^, the capacity was recovered to 1201.1 mAh g^−1^. On the contrary, the pure Si anodes suffer fast capacity decay as the current increases, and fail to deliver capacity when the current increases to 1.0 A g^−1^.

**Figure 8:**
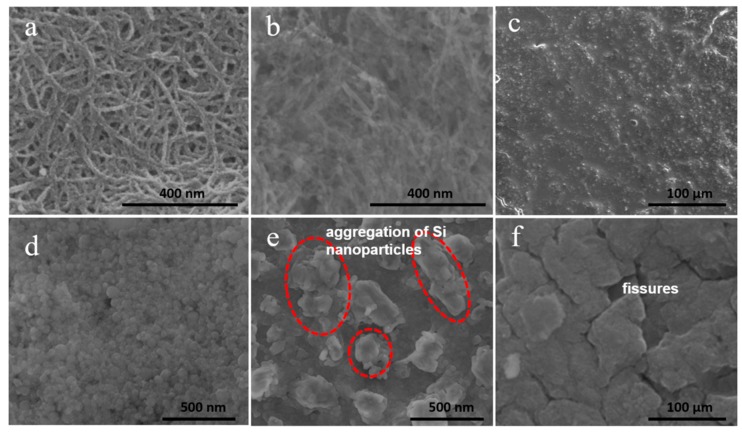
SEM images of Si/SiC/CNT composite before cycling (**a**) and after 100 cycles (**b**). SEM image of Si/SiC/CNT electrode after 100 cycles (**c**). SEM images of pure Si material before cycling (**d**) and after 100 cycles (**e**). SEM image of pure Si electrode after 100 cycles (**f**).

**Figure 9:**
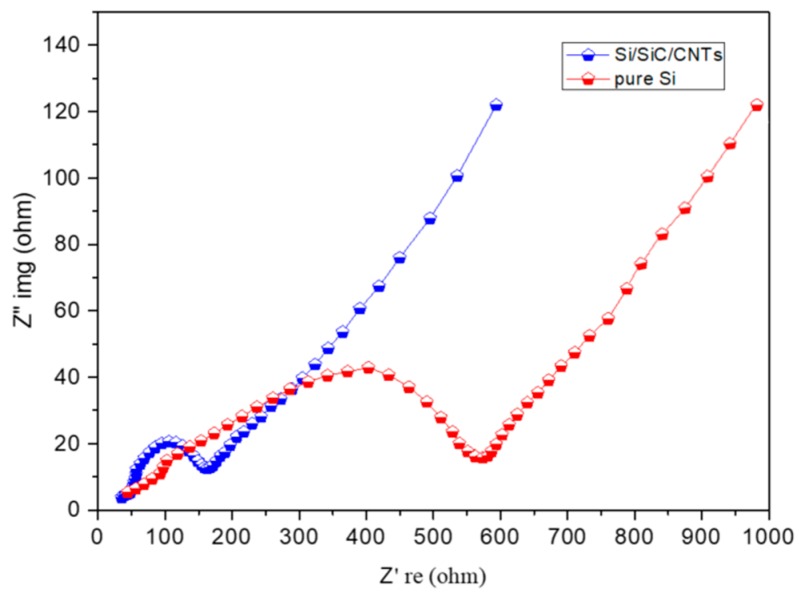
The EIS spectra of Si/SiC/CNT anode and pure Si anode after 100 cycles.

The improvement of electrochemical performance of Si/SiC/CNT nanocomposite could be attributed to the following effects. First, the SiC formed in the interfacial region between CNTs and Si can fix Si nanoparticles on the CNTs’ surface, avoiding detachment and electrochemical aggregation of Si nanoparticles. To study this positive effect of SiC, the morphological changes of Si/SiC/CNT composite and pure Si before and after 100 cycles was studied. SEM images show that Si/SiC/CNT nanocomposite after 100 cycles retains its original structure (Figure 8a) in which Si nanoparticles (~20 nm) remain on CNTs’ surface without detachment and aggregation (Figure 8b). As a contrast in Figure 8e, serious aggregations of Si nanoparticles after 100 cycles can be observed and their diameter is 200–700 nm which is much larger than that of initial Si nanoparticles (Figure 8d). Second, the good mechanical flexibility of CNTs could accommodate the volume expansion of Si nanoparticles during the lithiation process. Figure 8c,f shows the SEM images of the Si/SiC/CNT electrode and pure Si electrode after 100 cycles. Apparently, the pure Si electrode after 100 cycles exhibits large fissures and exfoliation (Figure 8f), which result from huge structural and volume changes of Si during cycling processes. In contrast, no disintegration can be observed for Si/SiC/CNT electrode after 100 cycles (Figure 8c). Third, CNTs with high electrical conductivity offer a conductive network which could promote electron transfer. This effect was studied by electrochemical impedance spectroscopy (EIS). Figure 9 exhibits the Nyquist plots recorded for the Si/SiC/CNT and pure Si half-cells after 100 cycles. The Rct of Si/SiC/CNT composite (130 Ω) was smaller than that of pure Si (490 Ω), indicating that CNTs obviously improve the electrical conductivity of a Si-based anode.

## 4. Conclusions

In summary, we developed a facile design and synthesis of Si/SiC/CNT nanocomposite based on the magnesium thermal reduction method. The Si/SiC/CNT nanocomposite exhibits a unique structure in which the Si nanoparticles obtained are uniformly distributed on CNTs’ surfaces. In addition, the SiC formed between Si and CNTs, serving as an interfacial layer that effectively stabilizes the Si nanoparticles and buffers the mechanical stress of Si nanoparticles, is caused by large volume expansion during discharge-charge process. Thus, the Si/SiC/CNT nanocomposite not only has a high initial reversible capacity of 1321.8 mAh g^−1^, but demonstrates a high capacity retention of 83.8% at 100 mA g^−1^ after 200 cycles. The Si/SiC/CNT nanocomposite can, therefore, be considered a promising anode material for LIBs and our simple and practical method could also be used to design other hybrid Si/C anode materials for LIBs.
